# Supplementary material for: Lobular Carcinoma of the Breast: A Comprehensive Review with Translational Insights
Source: Cancers (Basel). 2023 Nov 20;15(22):5491. doi: 10.3390/cancers15225491 (PMC10670219; doi:10.3390/cancers15225491)
Supplement: Supplementary file 1 [file cancers-15-05491-s001.zip › cancers-2662169-supplementary.pdf]

Supplementary Table S1:

Treatment schema in ILC

Early stage ILC treatment	*Salient points to note*
No specific guidelines	
Options include neoadjuvant/adjuvant chemotherapy or endocrine therapy	- Neoadjuvant is less effective in ILC compare to IDC (Section 9.1)<br>- adjuvant chemotherapy is effective in a subset of lobular carcinoma cases (clinically high-risk cases with gross lymph node involvement, larger tumor size and lymphovascular invasion) (Section 9.5)
Considerations for chemotherapy: molecular risk (grade, molecular tools like Oncotype DX or Mammaprint) and tumor burden (size and number of involved lymph nodes and menopausal status)	

Treatment in metastatic setting		
First line	Second line	Additional
i) Endocrine therapy +/- CDK4/6i<br><br>Abemaciclib (MONARCH 2,3)<br>Ribociclib (MONALEESA 2,3 AND 7)<br>Palbociclib (PALOMA 2,3)<br><br>ii) Endocrine therapy alone followed by CDK4/6i + ET (SONIA trial)	Alpelisib +ET (PIK3CA)	Standard chemotherapies
	PARPi (in gBRCA1/2, sBRCA, gPALB2 mutations)<br>Pembrolizumab (MSI-H/dMMR)	
	Everolimus+ Exmestane or Fulvestrant	
	Fulvestrant+ alternative CDK4/6i (Ribociclib or Abemaciclib)	Trastuzumab+ Deruxtecan (DESTINY-Breast 04)<br><br>Sacituzumab Govitecan (Tropics 02)
	Fulvestrant alone (less preferred)	
	Latest is Elacestrant in ESR1 mutated cases	
